# Are changes in sulfate assimilation pathway needed for evolution of C_4_ photosynthesis?

**DOI:** 10.3389/fpls.2014.00773

**Published:** 2015-01-13

**Authors:** Silke C. Weckopp, Stanislav Kopriva

**Affiliations:** Botanical Institute and Cluster of Excellence on Plant Sciences, Cologne Biocenter, University of Cologne, Cologne, Germany

**Keywords:** sulfate assimilation, C_4_ photosynthesis, bundle sheath cells, *Flaveria*, glutathione

## Abstract

C_4_ photosynthesis characteristically features a cell-specific localization of enzymes involved in CO_2_ assimilation in bundle sheath cells (BSC) or mesophyll cells. Interestingly, enzymes of sulfur assimilation are also specifically present in BSC of maize and many other C_4_ species. This localization, however, could not be confirmed in C_4_ species of the genus *Flaveria*. It was, therefore, concluded that the bundle sheath localization of sulfate assimilation occurs only in C_4_ monocots. However, recently the sulfate assimilation pathway was found coordinately enriched in BSC of *Arabidopsis*, opening new questions about the significance of such cell-specific localization of the pathway. In addition, next generation sequencing revealed expression gradients of many genes from C_3_ to C_4_ species and mathematical modeling proposed a sequence of adaptations during the evolutionary path from C_3_ to C_4_. Indeed, such gradient, with higher expression of genes for sulfate reduction in C_4_ species, has been observed within the genus *Flaveria*. These new tools provide the basis for reexamining the intriguing question of compartmentalization of sulfur assimilation. Therefore, this review summarizes the findings on spatial separation of sulfur assimilation in C_4_ plants and *Arabidopsis*, assesses the information on sulfur assimilation provided by the recent transcriptomics data and discusses their possible impact on understanding this interesting feature of plant sulfur metabolism to find out whether changes in sulfate assimilation are part of a general evolutionary trajectory toward C_4_ photosynthesis.

## INTRODUCTION

Sulfur is an important element in living organisms where it possesses a plethora of functions. As a component of the amino acids cysteine and methionine, sulfur is incorporated in peptides and proteins. The formation of disulfide bridges by cysteine residues of proteins is important for conformational and regulatory processes: however, sulfur in proteins possesses also catalytic and electrochemical functions and participates in electron transport in iron–sulfur clusters, is part of the catalytic sites of various enzymes and coenzymes. Furthermore, the sulfhydryl group of the tripeptide glutathione (GSH) is involved in redox reactions which protect the cell from oxidative stress (Leustek and Saito, 1999; Foyer and Noctor, 2009).

Plants have the ability to incorporate reduced sulfur components from the atmosphere in form of sulfur dioxide or hydrogen sulfide, but the majority of sulfur in higher plants derives from sulfate (Leustek et al., 2000; Saito, 2004). Sulfate (SO_4_^2–^) is present in the soil as the most oxidized and accessible form of sulfur for plants, algae and many microorganisms. Sulfate uptake into the plant and its distribution within the cells, occurs via various sulfate transporters which differ in their properties and functions (Leustek and Saito, 1999; Buchner et al., 2004; Takahashi et al., 2011). Within plant cells, sulfate can either be stored in the vacuole or directly be incorporated in organic compounds. For synthesis of reduced sulfur compounds, sulfate needs to be reduced and assimilated to cysteine. The initial step of sulfur assimilation is the activation of sulfate, an inert and stable compound, by ATP sulfurylase (ATPS) to adenosine-5′-phosphosulfate (APS). APS can be reduced to sulfite by APS reductase (APR) or phosphorylated to the common sulfo-group donor 3′-phosphoadenosine-5′-phosphosulfate (PAPS) by APS kinase. APR uses two molecules of reduced GSH for the reduction of APS. Sulfite reductase (SIR) then reduces sulfite to sulfide by transferring electrons from the iron–sulfur protein ferredoxin. Finally, sulfide can be incorporated in the amino acid backbone of *O*-acetylserine (OAS) by OAS (thiol)lyase (OASTL). OAS is derived from serine by acetylation mediated by serine acetyltransferase (SAT; Leustek et al., 2000; Saito, 2004; Kopriva, 2006). Serine biosynthesis is closely linked to carbon and nitrogen assimilation, the synthesis of cysteine thus merges the three assimilatory pathways. Accordingly, light, carbon, and nitrogen compounds regulate sulfate assimilation (Kopriva et al., 1999, 2002; Koprivova et al., 2000). Using thiol- and stress-dependent regulation of APR expression as a tool, the reduction of activated sulfate was found to be a key step in sulfur assimilation (Vauclare et al., 2002; Scheerer et al., 2010).

Reduced sulfur is preferentially stored and transported in the form of GSH, the most abundant low molecular weight thiol in plants with a number of different functions. It is substantially involved in the detoxification of reactive oxygen species (ROS) and heavy metals, serves as sulfur donor in catalytic processes and is involved in redox signaling (Foyer and Noctor, 2009; Takahashi et al., 2011). Two enzymes catalyze the ATP-dependent reactions to form GSH from its constituent amino acids. γ-glutamylcysteine synthetase (γ-ECS) catalyzes the reaction of cysteine and glutamate to form γ-glutamylcysteine (γ-EC). Subsequently, glycine is added to γ-EC by GSH synthetase to form GSH.

The traditional view on the whole plant sulfur metabolism suggests that sulfate reduction predominantly takes place in leaves and reduced sulfur compounds are subsequently distributed to sink tissues (Herschbach and Rennenberg, 2001). However, although GSH is widely used to store and transport reduced sulfur, most plant tissues are capable of sulfate reduction and thus are able to cover their needs of reduced sulfate. Indeed, according to available microarray data in the Genevestigator database, APR and SIR transcripts are present in all *Arabidopsis* tissues, including reproductive organs (Zimmermann et al., 2004). The presence of sulfate reduction could also be detected in roots and developing seeds (Brunold and Suter, 1989; Kopriva et al., 2001; Tabe and Droux, 2002).

There are, however, exceptions and some plant tissues seem to have lost the capacity to reduce sulfate. Most notable among these tissues are the mesophyll cells (MC) in C_4_ plants, as in these plants the pathway is confined to a specific tissue, the bundle sheath cells (BSC; Gerwick et al., 1980; Passera and Ghisi, 1982; Burnell, 1984; Schmutz and Brunold, 1984; Burgener et al., 1998). The intercellular distribution of sulfate (and nitrate) assimilation in C_4_ plants is one of the enigmatic open questions of sulfur metabolism, not just in terms of mechanisms but mainly of its biological significance. This question has been very recently revived by Aubry et al. (2014) who showed that genes involved in the pathway are mainly expressed in the BSC of *Arabidopsis*. Therefore, here we review what is known about the localization of sulfate assimilation in C_4_ plants, and discuss how the current progress in studies of C_4_ photosynthesis may contribute to understanding this interesting feature of plant sulfur metabolism.

## C_4_ PHOTOSYNTHESIS

During photosynthesis, carbon fixation takes place in the Calvin–Benson cycle and is mediated by the dual-specific enzyme Ribulose 1,5-bisphosphate carboxylase/oxygenase (Rubisco). The carboxylation reaction yields 3-phosphoglycerate (3-PGA) which is reduced to carbohydrates. In contrast, the oxygenation reaction yields 2-phosphoglycolate (2-PG), a toxic compound that enters photorespiration to be metabolized. The photorespiratory pathway releases one molecule of previously fixed CO_2_ and regenerates the other into 3-PGA. Although Rubisco shows higher affinity toward CO_2_ than O_2_, one third of the assimilated carbon is lost during photorespiration, increasing so the energy costs of photosynthesis. An efficient mechanism to minimize the oxygenation reaction of Rubisco and photorespiration is C_4_ photosynthesis. C_4_ plants avoid such loss of assimilated CO_2_ by splitting the CO_2_ fixing reactions among two differentiated cell types, MC and BSC. MC and BSC differ from each other by their morphological properties, distribution within the plant tissue and cell-specific localization of many enzymes. BSC build up a radial pattern around the vascular tissue. They exhibit thickened cell-walls and many starch containing chloroplasts. BSC are surrounded by an adjacent layer of MC with small and randomly distributed chloroplasts. The radial arrangement of BSC and MC around the vascular tissue is known as Kranz anatomy (Laetsch, 1974).

The primary CO_2_ fixation takes place in MC. This reaction, catalyzed by phosphoenolpyruvate carboxylase (PEPCase), results in oxaloacetate, a C_4_ compound. Oxaloacetate is immediately converted to malate or aspartate, C_4_ acids that enter BSC by diffusion. Here, the C_4_ acids are decarboxylated, resulting in enriched CO_2_ concentrations in the BSC. C_4_ photosynthesis can be divided into three subtypes defined by the enzymes which catalyze the decarboxylation reaction: NADP-dependent malic enzyme, NAD-dependent malic enzyme or phosphoenolpyruvate carboxykinase. The released CO_2_ enters the Calvin–Benson cycle within the BSC (Edwards and Huber, 1981; Edwards and Walker, 1983; Hatch, 1987). Besides Rubisco, additional genes involved in the carbon cycle are solely expressed in the BSC (Sheen, 1999; Edwards et al., 2001). The separation of PEPCase and Rubisco, as well as the Kranz anatomy and a lack of photosystem II (PSII) in BSC of most C_4_ plants (Hatch and Osmond, 1976) support more effective carbon assimilation. The CO_2_ enrichment in the BSC minimizes the rate of the oxygenation reaction. Any CO_2_, lost during residual photorespiration, is separated from the aerial space by the thick cell walls of the BSC and by the adjacent layer of MC, where it is directly re-assimilated. In addition, the lack of PSII in BSC restricts the emergence of oxygen by water splitting to MC chloroplasts. Paired with a BSC-specific expression of glycine decarboxylase (GDC), these mechanisms generate a low photosynthetic CO_2_ compensation point in C_4_ plants. The CO_2_ compensation point describes the concentration at which the photosynthetic CO_2_ uptake equals the rate of respiration.

The last decade has seen a revival of interest in C_4_ photosynthesis, as a potential route to increase crop productivity (Hibberd et al., 2008). Particular interest has been given the evolutionary route from C_3_ to C_4_ plants as this could identify steps needed to engineer C_4_ photosynthesis in C_3_ plants (Heckmann et al., 2013; Mallmann et al., 2014). C_4_ photosynthesis evolved not less than 62 times independently from C_3_ ancestors (Sage et al., 2011). Genera containing C_3_ and C_4_ as well as C_3_–C_4_ intermediate species, such as *Flaveria*, are especially suited to give information about the evolution from C_3_ to C_4_ photosynthesis (Kopriva et al., 1996; Heckmann et al., 2013; Williams et al., 2013; Mallmann et al., 2014). C_3_–C_4_ species are characterized by a CO_2_ compensation point intermediate of C_3_ and C_4_ species (Apel and Maass, 1981; Bauwe et al., 1987). While there are many different ways to achieve such CO_2_ compensation points (Bassüner et al., 1984; Bauwe, 1984; Monson et al., 1986), the underlying photosynthetic mechanism is universally based on a photorespiratory CO_2_ pump. Shortly, GDC activity is lost in the MC, resulting in the transport of photorespiratory glycine to BSC for decarboxylation, which causes a moderate increase in BSC CO_2_ concentration and a boost of carboxylation over oxygenation reaction. Since the two-carbon compound glycine serves as C transporter, this process is known as C_2_ photosynthesis (Bauwe and Kolukisaoglu, 2003; Sage, 2004; Sage et al., 2012; Schulze et al., 2013). C_2_ photosynthesis was postulated to be a prerequisite to C_4_ metabolism (Sage et al., 2012). This conclusion was confirmed by analysis of transcriptomes of nine *Flaveria* species and by flux balance analysis (Mallmann et al., 2014). However, the translocation of glycine decarboxylation from MC to BSC results in the shift of carbon and nitrogen balance. This disbalance can be counterbalanced by a basal activity of the C_4_ cycle, acting as an efficient ammonium recirculation pathway. Thus, the C_2_ photosynthesis triggers development of a basic C_4_ cycle which further leads to evolution of a full C_4_ photosynthesis. In another mathematical approach, a model of fitness landscape describes the evolutionary trajectory from C_3_ to C_4_ photosynthesis as a process of 30 individual steps, each of them gradually yielding a gain of biochemical fitness (Heckmann et al., 2013). This model positions the known C_3_–C_4_ intermediates as real transitory states in the evolution from C_3_ to C_4_ photosynthesis and explains the ease with which C_4_ photosynthesis evolved independently, making it feasible to be recreated by genetic engineering.

However, one major aspect of C_4_ photosynthesis remains largely unexplained. Besides the cell-specific distribution of enzymes involved directly in photosynthesis and photorespiration, intercellular differences could be detected in the localization of enzymatic reactions involved in nitrogen and sulfur assimilation in C_4_ plants. The reduction of nitrate and nitrite is restricted to MC, whereas the incorporation of reduced nitrogen into the amino acids glutamate and glutamine takes place in the BSC or in MC and BSC (Rathnam and Edwards, 1976; Moore and Black, 1979; Becker et al., 2000) which accords with the translocation of GDC into BSC. C_4_ species also exhibit higher nitrogen use efficiency, presumably because of the concentration of Rubisco into BSC and so decreasing the amount of this protein per leaf area (Brown, 1978; Ghannoum et al., 2011). Whether the cell-specific localization of nitrate reduction contributes to the improved nitrogen nutrition is not clear yet. Furthermore, since spatial separation was reported for the assimilation of carbon and nitrogen, two nutrients that are taken up into the plant in their oxidized form (CO_2_ and NO_3_^–^) and since the translocation of GDC to the BSC restricts production of photorespiratory serine, the precursor of OAS and cysteine, to these cells, the question of intercellular distribution of sulfate assimilation in C_4_ plants has long been of major interest.

## INTERCELLULAR COMPARTMENTATION OF SULFATE ASSIMILATION IN C_4_ PLANTS

**FIGURE 1:**
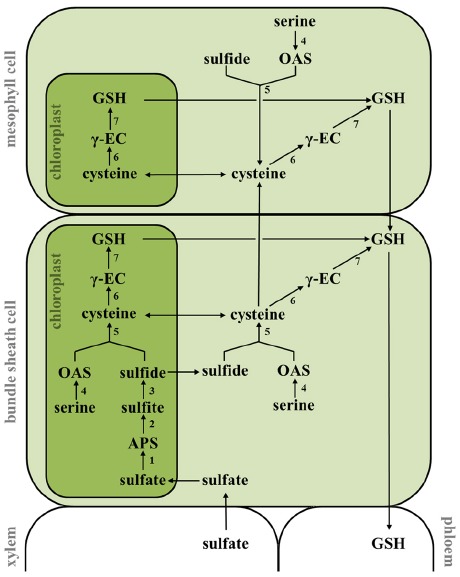
**Intercellular compartmentation of sulfate assimilation and glutathione biosynthesis in maize.** Sulfate is taken up from the soil and transported to the bundle sheath cells (BSC) through the xylem. The reduction of sulfate takes place exclusively in the plastids of BSC and is mediated by ATP sulfurylase (1), APS reductase (2), and sulfite reductase (3). Sulfide is further incorporated into the amino acid backbone of OAS by OAS (thiol)lyase (5) to form cysteine in chloroplasts, cytosol and mitochondria (not included) of BSC. OAS is derived from serine by serine acetyltransferase-mediated acetylation (4). Reduced sulfur is transported in form of cysteine to mesophyll cells where glutathione (GSH) synthesis is predominantly localized. GSH synthesis is driven by γ-EC synthetase (6) and GSH synthethase (7). APS, adenosine-5′-phosphosulfate; GSH, glutathione; OAS, *O*-acetylserine; γ-EC, γ-glutamylcysteine.

The question of intercellular compartmentation of sulfate assimilation in C_4_ species was first addressed by Gerwick and Black (1979) in the crabgrass *Digitaria sanguinalis*. They focused on the initial step of sulfur assimilation, the activation of sulfate by ATPS, and showed that more than 90% of total leaf ATPS activity was restricted to BSC in crabgrass. Similar results could be obtained for 17 additional C_4_ monocot species, representing each of the three C_4_ subtypes (Gerwick et al., 1980; Passera and Ghisi, 1982; Burnell, 1984; Schmutz and Brunold, 1984; Burgener et al., 1998). Analyses of the spatial separation of sulfate assimilation in maize leaves could additionally show restriction of APR activity to BSC (Figure 1; Schmutz and Brunold, 1984; Burgener et al., 1998), whereas activity of SIR and OASTL was equally detected in MC and BSC (Passera and Ghisi, 1982; Schmutz and Brunold, 1984, 1985).

Corresponding to these findings, transcripts of ATPS and APR were found exclusively in the BSC and OASTL mRNA was present in MC and BSC in maize leaves (Kopriva et al., 2001). These findings were supported by recent analyses of the maize MC and BSC transcriptomes which showed a predominant expression of all four ATPS as well as two APR genes in the BSC and localization of OASTL transcripts in MC and BSC (Chang et al., 2012). The identical localization of APR, ATPS, and OASTL mRNA and their enzymes activity, indicates a transcriptional regulation of genes involved in sulfate assimilation. However, APR mRNA but not enzyme activity could also be detected in MC of maize leaves after exposure to chilling stress. This finding reveals participation of post-transcriptional processes in the compartmentalization of sulfate assimilation in maize (Kopriva et al., 2001). Interestingly, post-transcriptional mechanisms seem to be responsible also for the MC-specific localization of glutathione reductase (GR) in maize (Pastori et al., 2000).

Although SIR activity was originally detected in MC of maize leaves (Schmutz and Brunold, 1984), in further studies SIR transcripts were shown to accumulate only in BSC (Kopriva et al., 2001; Chang et al., 2012). The localization of SIR, particularly the discrepancy in protein and transcript, thus needs revisiting, although it is possible that in MC SIR fulfills a role independent from sulfate assimilation, e.g., protection against SO_2_ (Yarmolinsky et al., 2013). Burgener et al. (1998) were able to show that all steps of sulfate reduction take place in BSC. The restriction of sulfate reduction to BSC requires an efficient transport of reduced sulfate compounds to the adjacent MC. Feeding experiments with [^35^S]sulfate showed that cysteine is the transport metabolite in maize. It is transported from BSC to MC where GSH synthesis is predominantly localized (Burgener et al., 1998).

The question of evolutionary significance of the BSC-specific localization of sulfate assimilation was addressed in the genus *Flaveria* (Koprivova et al., 2001). Comparing the cell-specific localization of ATPS and APR mRNA by northern analysis and *in situ* hybridization in various *Flaveria* species, Koprivova et al. (2001) expected an accumulation of the transcripts in the BSC in C_4_ species and ubiquitous expression of ATPS and APR in all photosynthetic cells of C_3_ species. Remarkably, they found comparable transcript levels of both genes in each species, independent of the photosynthetic mechanism. Immunogold electron microscopy confirmed a similar distribution of APR protein in chloroplasts of BSC and MC in all species analyzed (Koprivova et al., 2001).

These findings contradicted the previously postulated compartmentation of sulfate assimilation in C_4_ plants (Gerwick et al., 1980; Passera and Ghisi, 1982; Burnell, 1984; Schmutz and Brunold, 1984; Burgener et al., 1998). Admittedly, earlier studies were conducted in maize and 17 other C_4_ species, all belonging to the group of monocotelydons. *F. trinervia* and *F. australasica* were the first C_4_ dicots analyzed for the intercellular separation of sulfate assimilation (Koprivova et al., 2001). However, BSC-specific localization of sulfate assimilation is not a monocot-specific trait. In wheat, a C_3_ monocot, ATPS and APR activities were found at equivalent levels in MC and BSC (Schmutz and Brunold, 1984). In addition, recently, Aubry et al. (2014) described the preferential expression of genes associated with sulfate assimilation in the BSC of the C_3_ dicot *Arabidopsis thaliana*. Thus, BSC-specific localization of sulfate assimilation might no longer be seen as a C_4_-associated trait but possibly as species-specific characteristic. Therefore it is even more imperative to understand the biological reasons and/or consequences for this localization.

Indeed, the physiological significance of spatial separation in sulfate assimilation in maize has been widely discussed, unfortunately without a clear conclusion. The BSC of maize lack PSII and with it the water-splitting complex (Sheen and Bogorad, 1988; Pfundel et al., 1996). Burgener et al. (1998) considered that the consequential reduction in O_2_ concentration might be the reason for BSC-specific expression of sulfate assimilation in maize. The low levels of O_2_ might prevent the oxidation of sulfite and sulfide, the intermediates of sulfate assimilation, and so increase the efficiency of the pathway. However, such oxidative events, except the enzymatic sulfite oxidation by sulfite oxidase, have not been reported for C_3_ plants which possess PSII in the chloroplasts of all photosynthetic cells. As a precursor of cysteine, serine is necessary for sulfate reduction. In C_3_ plants, photorespiration is the main source of serine production (Douce et al., 2001; Ros et al., 2014). GDC and serine hydroxymethyltransferase, key enzymes in photorespiration and serine biosynthesis, are localized in BSC of C_4_ plants (Ohnishi and Kanai, 1983; Gardeström et al., 1985; Becker et al., 1993). Therefore, Burgener et al. (1998) hypothesized that the localization of sulfate reduction in maize coincides with the site of photorespiration because of serine availability. Several physiological aspects, however, challenge this hypothesis. C_4_ species of the genus *Flaveria* do not show BSC-specific localization of sulfur reduction, although GDC activity is restricted to this cell type. Moreover, serine would need to be transported from BSC to MC to participate, e.g., in protein biosynthesis. Indeed, an alternative pathway of serine biosynthesis exists and is vital for plants (Benstein et al., 2013), so that photorespiratory serine cannot be the link. The BSC localization of sulfate assimilation in *Arabidopsis* (Aubry et al., 2014) also contradicts the link to serine synthesis. The *Arabidopsis* data are derived from comparison of BSC expression vs. whole leaf expression, therefore the spatial separation may not be complete as it is in C_4_ monocots. The BSC expression of sulfate assimilation is linked with BSC expression of genes for synthesis of glucosinolates, sulfur-containing secondary metabolites. As these compounds are important in the vasculature for defense against insects (Shroff et al., 2008) the need for glucosinolate synthesis might drive the BSC specific expression of sulfate assimilation genes. This hypothesis, even though it has to be tested yet, seems to strengthen the view that the spatial distribution of sulfate assimilation may be a species-specific adaptation to diverse environmental conditions. Obviously, more research is needed to understand the biological reason(s) and consequences for plant fitness of such intercellular separation of sulfate assimilation, if there are any.

## SULFUR ASSIMILATION PATHWAY FROM C_3_ TO C_4_ PLANTS

The analysis of sulfate assimilation in different *Flaveria* species revealed another interesting result. The activity of APR as well as cysteine and GSH levels were significantly higher in leaves of C_4_ and C_4_-like species compared to C_3_ and C_3_–C_4_ species (Koprivova et al., 2001). Enzyme activity, cysteine, and GSH content correlated with the degree of expression of C_4_ characteristics, considering the photosynthetic CO_2_ compensation point as a quantitative measure of C_4_ photosynthetic traits (Kopriva and Koprivova, 2005). The intriguing results of analyses of sulfate assimilation in *Flaveria* were unfortunately not followed by further studies. The question of intercellular localization of sulfur assimilation, and nitrate assimilation at the same time, seemed to be forgotten for more than a decade. However, with the increased interest in C_4_ photosynthesis as a means to improve plant productivity and with the more frequent use of next generation sequencing in plant science, these questions can be addressed from a different angle.

**FIGURE 2:**
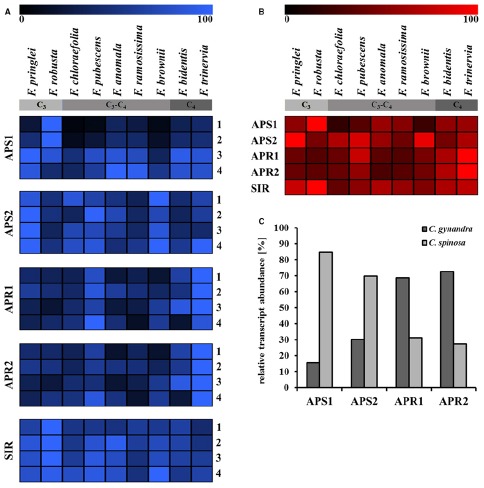
**Transcript abundance of genes involved in sulfate assimilation in leaves of C_3_, C_4_, and C_3_–C_4_ intermediate plants.** Based on the transcriptomic data of nine *Flaveria* species by Mallmann et al. (2014) obtained in four independent cultivations harvested at different seasons (1, September 2009; 2, June 2010; 3, October 2010; 4, April 2011), the transcript levels of the individual cultivations **(A)** and mean values **(B)** were normalized and plotted in heat maps. C_3_: *F. pringlei*, *F. robusta*; C_3_–C_4_: *F. chloraefolia*, *F. pubescens*, *F. anomala*, *F. ramosissima*; C_4_-like: *F. brownii*; C_4_: *F. bidentis*, *F. trinervia*. **(C)** The relative transcript abundances of genes participating in sulfate assimilation in the *Cleome* species *C. gynandra* (C_4_) and *C. spinosa* (C_3_) are based on the Brautigam et al. (2011). Transcripts were annotated by allocation to their *Arabidopsis* homologues. APR1 and APR2, APS reductase; APS1 and APS2, ATP sulfurylase; SIR, sulfite reductase.

Indeed, the transcriptomes of nine *Flaveria* species were analyzed recently to assess the influence of photorespiration on the evolution of C_4_ photosynthesis (Mallmann et al., 2014). To obtain the transcriptomic data, the authors performed four independent cultivations during different seasons of two C_3_, four C_3_–C_4_, one C_4_-like, and two C_4_ species of the genus *Flaveria* (Figure 2). The analysis showed clear gradients in expression of many genes across the *Flaveria* species, the photorespiratory enzymes being expressed to lower degree with increasing degree of C_4_ photosynthesis, whereas the genes of C_4_ pathway being expressed more. Data mining of this transcriptome dataset focusing on genes associated with sulfate assimilation provides an interesting insight into the expression of ATPS, APR and SIR, depending on the photosynthetic mechanism and seasonal changes (Figure 2A). APS1 and APS2 are two isoforms of ATPS in *Arabidopsis thaliana*, with transcript sequences matching *Flaveria* ATPS mRNA. Whereas APS1 is exclusively localized in chloroplasts, APS2 can also be found in the cytosol. The expression of the ATPS genes does not show a recognizable correlation with the photosynthetic mechanism, but seems to follow seasonal changes. Direct comparison of APS1 and APS2 signals in each species indicate preferential expression of one of the isoforms for at least the C_3_ species, *F. pringlei* and *F. robusta*, and the C_4_-like species *F. brownii* (Figure 2B). Overall, the highest expression of ATPS genes can be detected in *Flaveria* C_3_ species.

The transcript level of SIR was least variable among the different species and time points of harvest, with a tendency to higher levels in the C_3_ species (Figures 2A,B). This agrees with the low level of transcriptional regulation of this gene in the model species *Arabidopsis* (Takahashi et al., 2011). On the other hand, the differences in mRNA levels of genes similar to the *Arabidopsis* APR1 and APR2 sequences were striking (Figures 2A,B). Both isoforms show similar expression patterns, which indicates either a coordinate expression or a presence of a single APR isoform in the *Flaveria* species. Strongest expression of APR can be detected in the *Flaveria* C_4_ species *F. bidentis* and *F. trinervia*. The C_3_–C_4_ species *F. pubescens* constitutes an exception with rather high APR mRNA levels. The increased transcript levels of APR in C_4_ species correspond with the elevated APR enzyme activity in C_4_ and C_4_-like *Flaveria*, reported by Koprivova et al. (2001), and indicate transcriptional regulation of APR activity depending on the photosynthetic mechanism. To test whether this trait can be extended to other C_4_ species, we analyzed the comparative adult leaf transcriptomes of closely related C_3_ and C_4_ *Cleomaceae* species, *Cleome gynandra* (C_4_) and *Cleome spinosa* (C_3_; Brautigam et al., 2011). The ratio of ATPS and APR mRNA levels between the two species were comparable to transcript level relations in *Flaveria* C_3_ and C_4_ species (Figure 2C). The relative abundance of ATPS transcripts is higher in *C. spinosa*, whereas *C. gynandra* exhibits increased abundance of APR mRNA.

Adenosine-5′-phosphosulfate reductase has been reported to be the key enzyme of sulfate assimilation with strong regulation by many environmental factors (Kopriva et al., 2001; Vauclare et al., 2002; Scheerer et al., 2010). Among others, sulfur starvation and increased need for cysteine, required for GSH synthesis, induce APR transcription (Vauclare et al., 2002). Hence, increased transcript levels of APR in two independent C_4_ species compared to their close C_3_ relatives might indicate the need of enhanced levels of reduced sulfur in C_4_ plants. Indeed, C_4_ plants are particularly adapted to dry and warm habitats, environmental conditions which cause oxidative stress by formation of ROS. To protect the cells from damage, ROS is detoxified in the GSH–ascorbate cycle in which GSH serves as reductant of dehydroascorbate (Noctor and Foyer, 1998). Thus, exposure to oxidative stress increases the demand for GSH. Cysteine, required for GSH synthesis, is provided by sulfate reduction. Adaptation to exceptional habitats could explain the high levels of GSH and cysteine in C_4_ *Flaveria* described by Koprivova et al. (2001) as well as the increased APR activity and transcript levels. The C_4_ monocot maize is sensitive to chilling, another oxidative stress causing condition. Here as well exposure to chilling stress increases the mRNA levels and activity of APR and ATPS (Brunner et al., 1995; Kopriva et al., 2001). Remarkably, the species *Zea mays* also contains chilling-tolerant genotypes which show higher levels of GSH and cysteine as well as increased activity of APR and GR, the enzyme that reduces oxidized GSH (Kocsy et al., 1996, 1997). Kocsy et al. (2000) demonstrated a direct link between GSH synthesis and chilling tolerance by inhibiting γ-ECS using buthionine sulfoximine (BSO). BSO-treated maize plants lost their chilling tolerance which could be restored by supplementation with GSH or γ-EC.

Overall, these findings in maize fit the general hypothesis of demand-driven regulation of sulfate assimilation formulated from studies with C_3_ Brassicaceae species (Lappartient and Touraine, 1996). Sulfur starvation leads to increased transcript levels of ATPS, APR, and sulfate transporters in maize (Bolchi et al., 1999; Hopkins et al., 2004), whereas supplementation of reduced sulfur compounds results in repression of ATPS expression (Bolchi et al., 1999). Besides oxidative stress, cadmium has been reported to enhance ATPS and APR activity (Nussbaum et al., 1988). The detoxification of heavy metals requires the synthesis of phytochelatins which bind to the metals and enable their relocation to the vacuole. As GSH is indispensable for the formation of phytochelatins (Cobbett and Goldsbrough, 2002), exposure to heavy metals increases the demand for reduced sulfur. In Brassicacea species GSH was identified as the molecular regulator of demand-driven sulfate assimilation (Lappartient and Touraine, 1996; Vauclare et al., 2002). In contrast, cysteine directly regulates sulfate reduction in maize (Bolchi et al., 1999). Kopriva and Koprivova (2005) discussed a possible molecular background to the findings of Bolchi et al. (1999). They assumed that cysteine as regulatory molecule is a consequence of the BSC-specific localization of sulfate assimilation in maize. Whereas GSH can be synthesized in maize BSC and MC, cysteine synthesis is restricted to BSC and cysteine was shown to be transported from BSC protoplasts (Burgener et al., 1998; Gomez et al., 2004). This led to the conclusion, that the cysteine pools of MC and BSC are permanently connected and enable a rapid reaction to changes in cysteine concentration (Kopriva and Koprivova, 2005).

Regulation of sulfate assimilation in different *Flaveria* species has not been addressed yet. The elucidation of such regulation might give an insight view on the molecular mechanism causing a constitutionally higher activity of the sulfate assimilatory pathway in C_4_ species compared to C_3_ plants.

## CONCLUSION AND OUTLOOK

The BSC localization of sulfate assimilation and the expression gradient from C_3_ to C_4_ species are interesting aspects of both sulfur metabolism and C_4_ photosynthesis. It is important to find out whether this localization and difference in expression give the plants an evolutionary advantage, which has to be considered in attempts to engineer C_4_ photosynthesis in C_3_ crops as well as to improve sulfur use efficiency of crop plants. The wealth of genomics and transcriptomics resources in C_4_ plants offers new ways to approach these questions, based not on a single model. The findings of preferential localization of sulfate assimilation in BSC in *Arabidopsis* could be the right trigger to assess the consequences of this localization for the general sulfur metabolism. All in all, while we cannot answer the question of importance of the changes in expression and localization of sulfate assimilation for evolution of C_4_ photosynthesis yet it seems that this enigmatic question of plant sulfur research might be answered in near future.

### Conflict of Interest Statement

The authors declare that the research was conducted in the absence of any commercial or financial relationships that could be construed as a potential conflict of interest.
